# Structural, Photophysical, and Distributed Feedback Lasing Characteristics of Furan-Substituted Thiophene/Phenylene Co-Oligomer Single Crystals

**DOI:** 10.1021/acsami.5c05119

**Published:** 2025-06-16

**Authors:** Periyasamy Angamuthu Praveen, Thangavel Kanagasekaran, Chaoyan Ma, Masahiro Terada, Tienan Jin, Yusuke Wakabayashi, Hidekazu Shimotani

**Affiliations:** † Department of Physics, Graduate School of Science, 13101 Tohoku University, Sendai 980-8578, Japan; ‡ Organic Optoelectronics Laboratory, Department of Physics, 443874 Indian Institute of Science Education and Research - Tirupati, Tirupati 517619, India; § Department of Chemistry, Graduate School of Science, 13101 Tohoku University, Sendai 980-8578, Japan; ∥ Research and Analytical Center for Giant Molecules, Graduate School of Science, 13101 Tohoku University, Sendai 980-8578, Japan

**Keywords:** organic semiconductor lasers, optical pumping, DFB laser, gain narrowing, PLQY

## Abstract

A furan derivative, 2-[1,1’-biphenyl]-4-yl-5-(5′-[1,1’-biphenyl]-4-yl-[2,2’-bithiophen]-5-yl)­furan (BPFTT), was designed and synthesized and shown to have excellent optical and electronic characteristics. The synthesized compound exhibits a photoluminescence quantum yield (PLQY) of 28%. In addition, a field-effect transistor fabricated using this material in the active layer exhibits ambipolar mobilities of 0.54 (hole) and 0.03 (electron) cm^2^ V^–1^ s^–1^. Single crystals with their natural crystal edges acting as Fabry–Pérot resonators exhibit dual gain narrowing phenomena with optical pumping thresholds as low as 15 μJ/cm^2^. Introducing planar distributed feedback resonators reduces the threshold to 2.41 μJ/cm^2^, with the *Q*-factor reaching 1.5 × 10^4^. The full width at half-maximum is measured to be 0.074 nm, which is an excellent value reported to date for any organic single-crystal laser.

## Graphical abstract

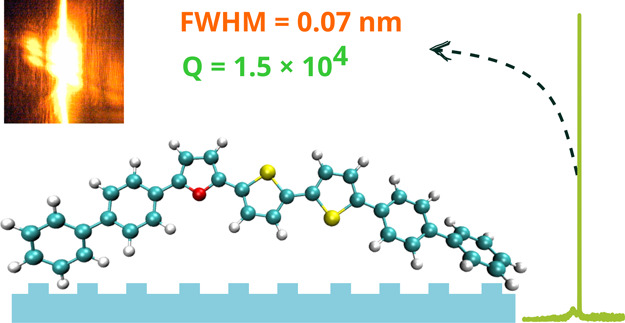

## 1 Introduction

Organic lasers are envisaged as a promising replacement for conventional solid-state lasers. Such requirements arise due to the band engineering difficulties in inorganic systems, making them hard to tune to a wavelength of interest. On the other hand, due to their tailorable structure and ease of processability, organic semiconductors (OSCs) can be designed to cover a wide color gamut and be fabricated literally on any substrate, making them an attractive candidate for flexible displays.^1^ Such a prospect has already been demonstrated in the case of organic light-emitting diodes. However, the realization of electrically pumped organic lasers is still a holy grail in optoelectronics. A potential lasing medium should have high mobility and photoluminescence quantum yield (PLQY).^2^ Nevertheless, both are contradicting phenomena in OSCs as higher mobility requires extended π-conjugation in the system, leading to significant luminescence quenching. In addition, to electrically pump an OSC laser, the active medium should be capable of withstanding current densities of approximately 10 kA/cm^2^.^1,3^ Most OSCs cannot withstand such extreme current densities, and so far, only a handful of materials have been demonstrated to be capable of lasing.^4–6^ As such, the synthesis of new materials and the evaluation of their optoelectronic properties are of paramount importance for identifying a suitable system.

From a materials design perspective, the molecule should have high oscillator strength with transition dipole moments aligned parallel to the molecular long axis.^7^ It should also be planar and rigid to reduce the number of nonradiative decay paths.^3,8^ The widely accepted strategy is to design and synthesize a material and evaluate its optically pumped lasing characteristics, particularly thresholds, to estimate its suitability for an electrically driven operation. With this approach, design principles can be integrated to achieve a minimal threshold with better stability.

In this regard, thiophene/phenylene co-oligomers (TPCO) are a widely studied molecular family for organic lasing.^9^ Furthermore, these materials can be fabricated in either a diode or a transistor architecture. Nevertheless, organic light-emitting transistor (OLET)-based single crystalline (SC) devices are advantageous for exploring the structure–property relationships and eliminating the need for an additional transistor to drive the device in the case of displays.^10^ In our recent work, we demonstrated the possibility of electrically driven lasing in a well-known TPCO, 5,5′-bis­(4-biphenylyl)-2,2’:5′,2’-terthiophene (BP3T), in the form of OLETs.^6^ Limitations such as strong singlet–triplet annihilation in BP3T can be reduced by substituting thiophene with moieties such as furan.^11,12^

Furan is an oxygen analogue of thiophene and has been shown to exhibit strong fluorescence characteristics suitable for organic lasing. Due to the lower atomic number of oxygen, furan does not suffer from heavy atom effects compared to thiophene.^13^ Incorporation of furan often results in smaller torsional angles, better molecular planarity, and shorter π–π stacking distances.^14^ Therefore, substituting thiophene with furan would improve the molecular rigidity and can tune the optical properties in the OSCs.^15,16^ Pertaining to this, recently, we have also shown the potential of central ring furan substitution in BP3T, resulting in a new derivative 2,5-bis­(5-[1,1’-biphenyl]-4-ylthiophen-2-yl)­furan (BPTFT) with excellent optical characteristics and improved planarity.^17^ In contrast, substitutions of furan do not consistently improve the optoelectronic properties of these compounds. In particular, depending on the substitution position, it can significantly affect charge transfer properties, such as mobility.^18^

In the present case, we have substituted furan for one of the terminal thiophene rings between the biphenyl and the central thiophene ring and synthesized 2-[1,1’-biphenyl]-4-yl-5-(5′-[1,1’-biphenyl]-4-yl-[2,2’-bithiophen]-5-yl)­furan (BPFTT), a derivative of BP3T. Such substitution does not alter the planarity throughout the molecule but only a part of the system. The reduced molecular planarity often weakens the π–π interaction in the solid state and limits the charge transfer mobility. We observed an approximately 2-fold reduction in hole mobility and a 3-fold reduction in electron mobility in the BPFTT system compared to BPTFT and BP3T. Despite this limitation, it exhibits excellent lasing characteristics and lower optical pumping thresholds than its counterparts. However, materials with lower mobilities are prone to thermal degradation at higher current injection densities, which limits their suitability for organic lasing. The usefulness of such materials is dependent on device engineering, such as mitigating the charge injection barrier, eliminating interfacial traps, and using additional feedback mechanisms to lower the lasing threshold.

Herein, by incorporating an additional planar resonator, we have shown that the threshold and required current density to achieve population inversion in an electrically driven BPFTT device can be reduced multifold. Thus, the inherent molecular limitations are compensated by device engineering concepts. Even though the potential of incorporating such gratings into organic light-emitting diode-based lasers has been demonstrated already, it has seldom been explored in the case of SC-OLET devices. We have shown that introducing planar distributed feedback (DFB) gratings dramatically improves the device performance through superior feedback and confinement in the cavity, resulting in excellent lasing characteristics. The results highlight the potential of combining molecular design with device engineering to address the limitations in realizing electrically driven single-crystal lasers and optimize their performance.

## 2 Results and Discussion

### 2.1 Synthesis

To synthesize the title compound, a mixture of 5-(biphenyl-4-yl)-5′-bromo-2,2’-bithiophene (**S1a**, 4.7 mmol, 1.87 g), (5-(biphenyl-4-yl)­furan-2-yl)­boronic acid (**S1b**, 7.0 mmol, 1.85 g), Pd­(PPh_3_)_4_ (0.235 mmol, 272 mg), and Na_2_CO_3_ (23.5 mmol, 2.5 g) was added to a mixture of THF and H_2_O (v/v = 2/1, 0.08 M, 60 mL) under a N_2_ atmosphere (Scheme 1). The reaction mixture was stirred for 48 h at 80 °C and then filtered by washing with water, MeOH, and CH_2_Cl_2_. The residue was further heated in CHCl_3_ at 80 °C and filtered while hot. The resulting green solid was further recrystallized in 1,2,4-trichlorobenzene at 200 °C (0.5 h) to give **BPFTT** in 64% yield (yellow solid, 1.6 g). Anal. Calcd for C_36_H_24_OS_2_: C 80.56, H 4.51, S 11.95. Found: C 80.63, H 4.59, S 12.04%. The resulting **BPFTT** was further purified by vacuum sublimation (∼5 × 10^–3^ Pa for more than 80 h) twice to obtain the material for crystal growth.

**Scheme 1:**
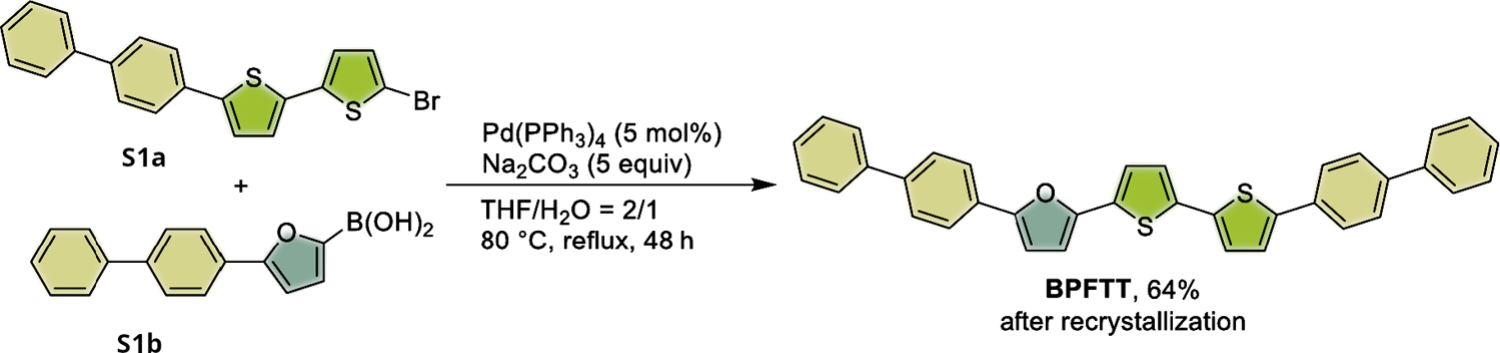
Synthesis Scheme for BPFTT

### 2.2 Structural Analysis

BPFTT SCs were grown by using physical vapor transport (PVT). A photomicrograph of grown crystals under UV irradiation is given in Figure 1a. Similar to its parent system BP3T, BPFTT crystallizes in orthorhombic symmetry, and the unit cell parameters are *a* = 5.66 Å, *b* = 7.53 Å, *c* = 59.04 Å, and *V* = 2517 Å^3^ for BPFTT. However, even after multiple attempts, the molecular structure was not solved. Like most other TPCOs, the BPFTT structure is also confirmed through mass spectroscopy (Figure S1).^19^ The structure was further studied using density functional theory (DFT) calculations (see Section 4), and the optimized geometry is given in Figure 1b.

**Figure 1:**
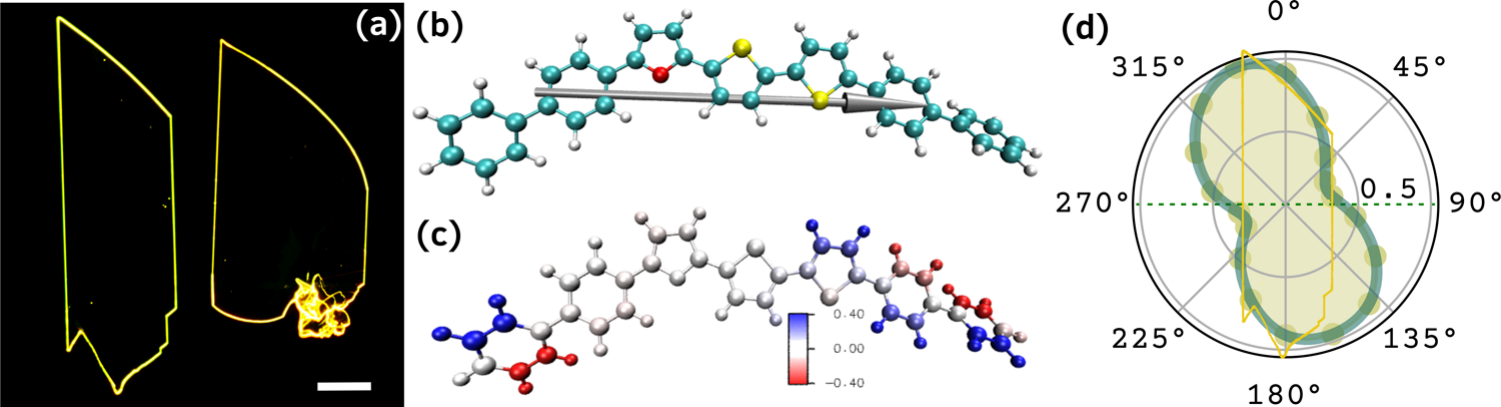
(a) Photomicrograph of PVT grown SCs at a sublimation temperature of 340 °C. Scale bar (white): 0.1 mm. (b) Optimized geometry (B3LYP/def2-SVP level) with the TDM projection from the excited-state analysis. (c) Molecular planarity plots for BPFTT and (d) PL intensity (560 nm) as a function of the polarization of the incident laser (5 mW, 405 nm). Dotted green line indicates the direction of polarization of the incident beam aligned parallel to the short axes of the BPFTT SCs.

It can be seen that the phenyls at both ends are slightly twisted from the plane of the thiophene–furan core. This explains the slight increase in crystalline volume in the case of BPFTT compared to its sister system, BPTFT. The bond length between the phenyl and furan cores is 1.456 and 0.010 Å shorter than that between the adjacent thiophene–phenyl cores. The bond length between the central thiophene and furan core is also 0.007 Å shorter than the thiophene–thiophene bond length. This indicates that the substitution of furan at the end increases the rigidity at only that part, opposing the case of BPTFT, where the rigidity is reflected throughout the system. This can be further analyzed by using the molecular planarity maps in Figure 1c. Blue and red regions indicate the fragment deviation in positive and negative directions with respect to the fitting plane (white).

The calculated molecular planarity parameter (MPP) and span of deviation from the plane (SDP) for BPFTT are 0.61 and 3.20 Å, respectively (see Section 4 and SI. Section 2). The gas-phase MPP and SDP of BPTFT are 0.43 and 1.77 Å and for BP3T, they are 0.78 and 3.78 Å, respectively, which indicate that the end-substitution slightly improves planarity for BPFTT compared to BP3T. The polarization-dependent photoluminescence (PL) of BPFTT SC is given in Figure 1d. The corresponding degree of linear polarization for the grown crystals is calculated to be 0.52 (SI. Section 3), which is slightly higher than the BPTFT system (0.47), indicating improved anisotropy and stronger emission directionality in the BPFTT SCs.^20,21^ Furthermore, the maximum emission was observed at the edges because the TDMs aligned horizontally along the width direction. The theoretically calculated transition dipole moment (TDM) for the S_1_ → S_0_ transition is given in Figure 1b. Like many other TPCO derivatives, the TDM is oriented along the molecular long axis, indicating that the molecules should be arranged perpendicular to the top and bottom surfaces of the crystal to obtain emission through edges.

### 2.3 Photophysical Properties

The electronic properties of the BPFTT system were analyzed using absorption, emission, and photoelectron yield spectroscopy (PYS) and are shown in Figure 2. From PYS, the highest occupied molecular orbital (HOMO) level is determined to be *E*_HOMO_ = −5.13 eV. The optical band gap (*E*_opt_) calculated from the Tauc plot is 2.15 eV (SI. Section 4, Figure S2). Based on this, the lowest unoccupied molecular orbital (LUMO) level and exciton binding energy are calculated to be 0.71 and −2.27 eV, respectively, which are only slightly different from those of the BPTFT system. The theoretically simulated HOMO and LUMO projections are given in Figure 2a. These frontier orbitals contribute almost ∼97% to the S_0_ → S_1_ transition. The LUMO level indicates a slightly lower charge injection barrier for calcium contacts in the case of ambipolar transistors compared to that of their counterparts. The recorded emission profiles for BPFTT SCs are given in Figure 2c. Most of the TPCOs are H-aggregates, and due to Davydov splitting, the 0 → 0 band is either forbidden or weakened. The emission peaks at 560 and 600 nm are therefore assigned to the 0 → 1 and 0 → 2 transitions, respectively. Corresponding Commission Internationale deI‘Eclairage (CIE) coordinates are (0.37, 0.62) and (0.62, 0.37), respectively, as shown in Figure 2d. The external PLQY for powdered BPFTT is 28%, almost equivalent to BPTFT (29%) and higher than that of BP3T (24%).

**Figure 2:**
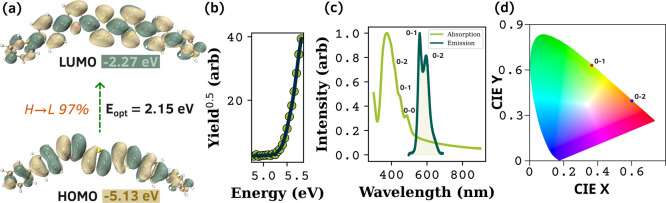
(a) Frontier molecular orbitals of BPFTT from the DFT (B3LYP/def2-SVP level) analysis. (b) PYS spectrum. (c) Normalized absorption and PL spectra. (d) CIE 1931 coordinates of BPFTT SC.

The photophysical properties are calculated experimentally using the Strickler–Berg relation (SI. Section 6).^22^ The obtained fluorescence lifetime and fluorescence rate constants for 0 → 1 and 0 → 2 transitions are 0.26 ns, 9.2 × 10^8^ s^–1^ and 0.36 ns, 7.6 × 10^8^ s^–1^, respectively. The values indicate the potential for the stimulated emission in both the bands of BPFTT.^23,24^ In organic systems, the characteristics of the excited state are influenced by the Franck–Condon (FC) and Herzberg–Teller (HT) states. To further investigate this, the excited-state dynamics of BPFTT were analyzed theoretically using time-dependent (TD)-DFT calculations. The theoretical fluorescence rate constant obtained for the singlet state is 2.35 × 10^10^ s^–1^, with approximately 97% of the contribution coming from HT states. This indicates a strong influence of vibrational coupling in the relaxation of BPFTT.

According to Kasha’s rule, the *S*_1_ state plays a dominant role in the emissive behavior of organic systems. To further explore the excitation dynamics of BPFTT, hole–electron analysis (HEA) was carried out for S_0_ → S_1_.^25,26^ The obtained HEA distribution and the fragment contribution heatmap are listed in Figure 3. Even though S_2_, S_3_, S_5_, and S_9_ states contribute to the process, their role is minimal (SI. Section 7). The excitation process is due to local excitons, i.e., both hole and electron occupy spatially similar regions. Fragment-wise, end phenyls have a minimal role to play in the excitation and relaxation process. On the other hand, furan’s contribution to electron distribution is significantly less than that of the thiophene core. Although similar to the case of BPTFT, the uneven distribution in the case of BPFTT affects its charge transport properties.

**Figure 3:**
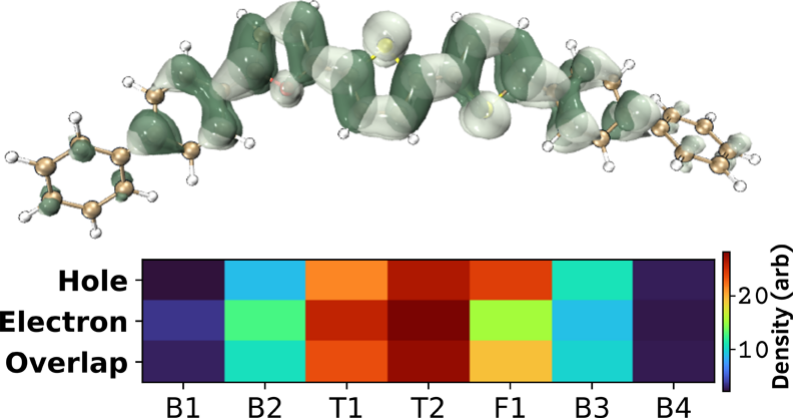
Hole–electron distributions of BPFTT from the excited-state DFT (B3LYP/def2-SVP level) analysis. The heatmap (a.u.) shows the molecular fragment contribution, where B_*n*_ is phenyl, T_*n*_ is thiophene, and *F*1 is furan.

### 2.4 Transistor Characteristics

The charge transport properties of BPFTT are analyzed in an organic light-emitting transistor (OLET) architecture, and a schematic of the device is shown in Figure 4a. Bottom-gate, top-contact devices are fabricated using asymmetric contacts (Figure 4b), and both p-channel and n-channel characteristics are studied. The transfer and output curves are obtained for both cases and are given in Figure 4c–f. The gate voltage (*V*_g_) was kept constant in the output measurement with an increasing drain voltage (*V*_d_). In the case of transfer curve measurement, *V*_d_ was kept at ±160 V, and *V*_g_ was varied. In OLETs, the recombination zone can be altered by modulating the *V*_g_, and the corresponding variation in the emission region was monitored using a top-mounted camera, as shown in Figure 4g. At high *V*_g_ (±60 V), the channel switches to unipolar operation due to the charge trapping with respect to *V*_g_ polarity. The obtained threshold values from the ${\sqrt{I}}_{d}$ versus *V*_g_ curves are 34 (n-drive) and −45 (p-drive) V, respectively. The BPFTT devices exhibit ambipolar characteristics with hole and electron mobilities of 0.54 and 0.03 cm^2^ V^–1^ s^–1^, respectively. The mobilities are significantly lower than those of the BPTFT and BP3T systems. However, these values are still better than some recently reported OSCs like BMeF and LD-1 for organic lasing.^27,28^ According to Marcus’ theory, two parameters, the reorganization energy (RE) and the charge transfer integral (CTI), contribute to the charge transfer mobility in organic semiconductors. The higher the CTI and the smaller the RE, the better the mobility. The DFT-calculated reorganization energy of BPFTT is 1220 cm^–1^, almost equivalent to that of BPTFT (1252 cm^–1^). On the other hand, a significant reduction in planarity would affect the molecular orbital overlap and decrease the charge delocalization.^29,30^ This, in turn, reduces both the CTI and mobility in the case of BPFTT. Nevertheless, contact engineering approaches, such as incorporating metal-oxide interlayers or innovative approaches, such as our previous work, can further improve the mobility.^31^

**Figure 4:**
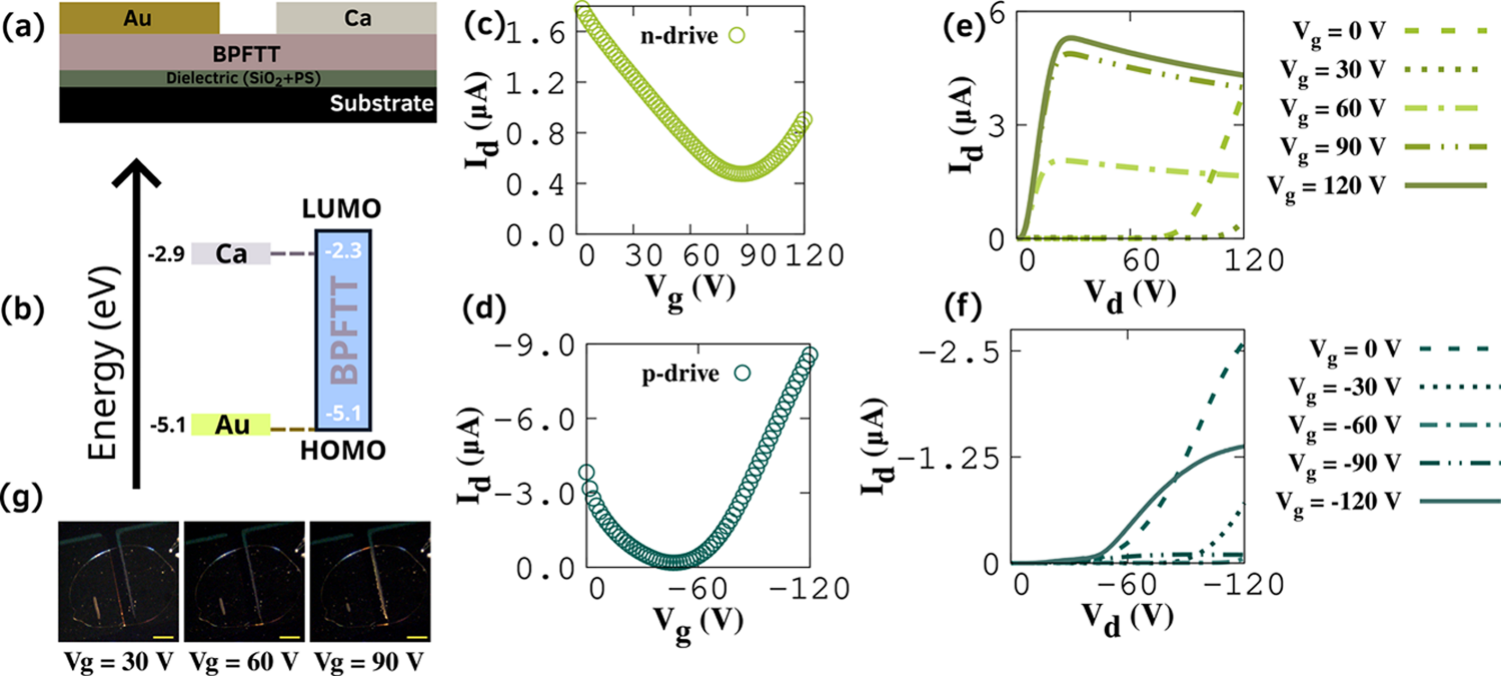
OLET characteristics of BPFTT measured in an Ar atmosphere inside a glovebox at room temperature. (a) Device structure. (b) Energy level diagram between BPFTT and metal contacts. Transfer curves for (c) n- and (d) p-drives. Output curves for (e) n- and (f) p-drives. (g) Electroluminescence from the device for different *V*_g_ (scale bar (yellow): 300 μm).

### 2.5 Lasing Characteristics

The crystal edges of BPFTT serve as the boundaries of natural Fabry–Pérot (FP) resonators, and the resulting cavity amplifies light upon external pumping through multiple internal reflections between the edges. Dominant emission peaks vary with respect to the thickness of the crystal. For SCs with thicknesses below 600 nm, the 0–1 band gain often narrows, and for thicknesses above 1 μm, the 0–2 band gain narrows. For SCs with a thickness between 600 and 1000 nm, both peaks often gain narrows, a phenomenon similar to that of its sister compound BPTFT.^17^ Hence, it is possible to modify the emission wavelength by tuning the crystal thickness (SI Section 11). Two crystals with thicknesses of approximately 600 and 800 nm are used for measuring 0–1 and 0–2 emission bands. At input intensity below the threshold, both BPFTT SCs exhibit a broad emission spectrum. Exceeding a threshold of 15 μJ/cm^2^ (0–1) and 17 μJ/cm^2^ (0–2), the dominant peak gain narrowing with a sudden reduction in full width at half-maximum (fwhm) indicates the amplified spontaneous emission (Figure 5a,b). Beyond the threshold, the fwhm varies only around 1 nm following the Schawlow–Townes formula. The observed thickness-dependent gain narrowing behavior is likely influenced by two competing factors: the wavelength-dependent confinement efficiency of optical modes and self-absorption. In thinner crystals (thickness <800 nm), optical modes associated with the 0–1 transition, having shorter wavelengths, are expected to exhibit higher confinement efficiency, while those of the 0–2 transition may be less effectively confined. This difference could favor the gain narrowing of the 0–1 transition. In contrast, in thicker crystals (thickness >800 nm), both transitions are likely to be well confined; however, the significant spectral overlap between absorption and emission near 560 nm leads to pronounced self-absorption of the 0–1 band, thereby favoring the 0–2 transition. Although this interpretation is consistent with the experimental trends,^27,32^ it remains speculative due to the lack of direct evidence. Further investigations are necessary to fully elucidate the underlying mechanism.

**Figure 5:**
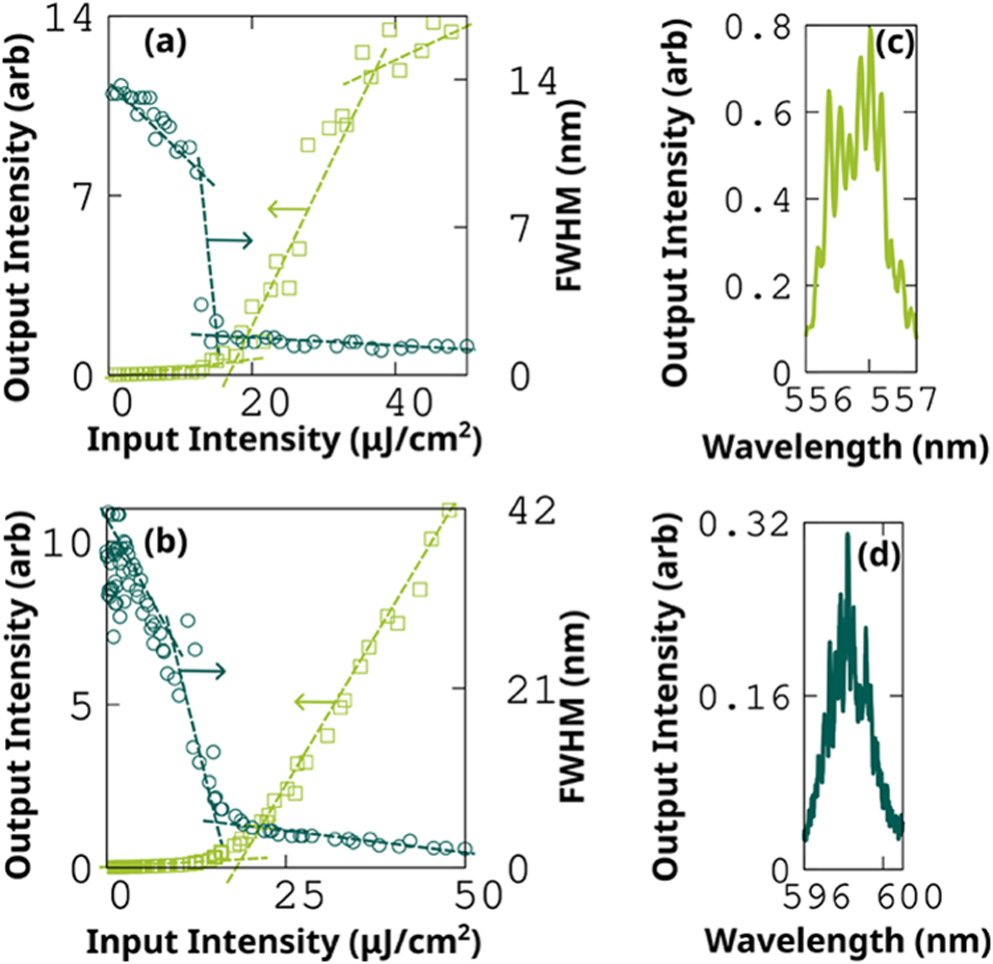
Lasing characteristics of BPFTT SC, pumped using a 337 nm UV laser at 13.8 Hz. (a, b) Gain narrowing curves for 0–1 and 0–2 bands obtained using a grating of 150 lines/mm. (c, d) PL spectrum obtained using high-resolution grating (1800 lines/mm) for 0–1 and 0–2 bands.

The calculated net gain coefficients (g) (see Section 4 and Figure S5) is 152 cm^–1^and 130 cm^–1^ for the 0–1 and 0–2 bands, respectively, at an excitation energy of 27.8 μJ/cm^2^. The corresponding loss coefficients (k) are 21 and 46 μJ/cm^2^ for the 0–1 and 0–2 bands, respectively. The calculated stimulated emission cross-section σ­(λ) values are 1.9 × 10^–16^ and 0.9 × 10^–16^cm^2^ for the 0–1 and 0–2 bands (SI. Section 12).^33^ Higher resolution emission spectra beyond the threshold show several distinct peaks that arise due to the longitudinal modes of the FP cavity (Figure 5c,d). For the crystal lateral widths 0.2 mm (0–1) and 0.3 mm (0–2), the obtained mode spacing values are 0.17 and 0.26 nm. The corresponding effective group refractive indices are calculated to be 5.05 and 2.44, respectively.

The fwhm (Δλ) obtained at a pump power of 47.8 μJ/cm^2^ for the 0–1 and 0–2 bands are 1.35 and 2.2 nm, respectively. Based on the fwhm, the calculated values of longitudinal coherence (SI. Section 13) are 36 and 25 μm, respectively. From this, temporal coherence was calculated to be 123 and 84 fs, respectively. The *Q*-factor is calculated using *Q* = λ/Δλ, where λ is the emission wavelength. The obtained *Q*-factors for the 0–1 and 0–2 bands are 415 and 268. Such moderate values of coherence and *Q*-factors can be attributed to the losses inside the cavity and the quality of feedback from the crystal edges; however, it is worth noting that the values are still better than those of the BPTFT system.

The optical threshold can be a measure to identify the required threshold for the electrically driven operation.^34^ Approximately 17 kA/cm^2^ of current density is required to realize an electrically driven laser (in the 0–2 band) without considering any losses. One way to reduce the threshold and device stability is by incorporating planar gratings, such as distributed feedback (DFB) resonators. In the present work, we fabricated gratings of period 140 nm using thermal nanoimprinting (see Section 4). The depth of the fabricated gratings measured using an atomic force microscope was 20 nm (Figure 6c). The BPFTT SCs are laminated on top of this, and the lasing characteristics are analyzed. At a threshold of 2.41 μJ/cm^2^ (SI. Figure S8), the broad emission spectrum shows a sudden increase in intensity with a fwhm of ∼1 nm (Figure 6a). Further increase in power improves the line width, and at a pump power of 8.93 μJ/cm^2^, the HR spectrum obtained using 1800 lines/mm grating exhibits a line width of 0.07 nm (Figure 6b), which is the narrowest for any single crystalline lasers reported so far (see Table 1). The current density required to achieve the population inversion in the case of the DFB device is calculated to be ∼4 kA/cm^2^ four times less than the FP device and the estimated Q-factor is about 1.5 × 10^4^.

**Figure 6:**
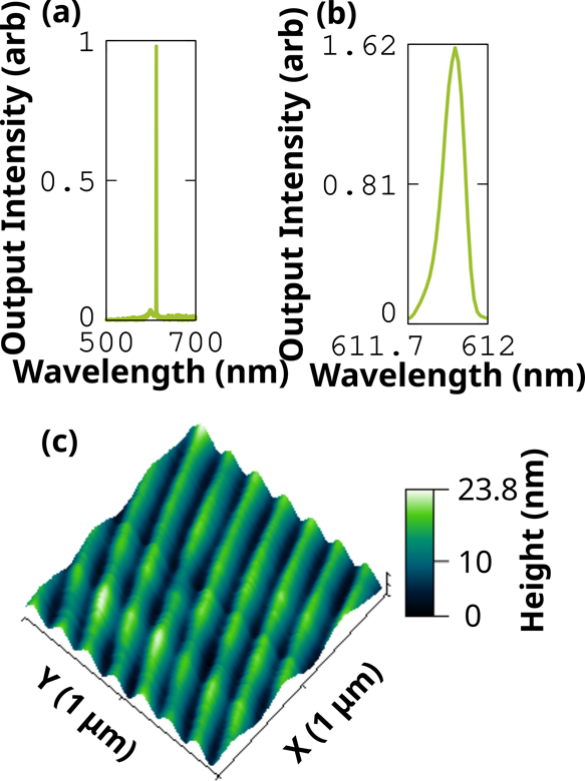
DFB lasing characteristics of the BPFTT SC. (a) PL spectra at the fluence of 4.06 μJ/cm^2^ (above the threshold). (b) High-resolution emission spectrum (1800 lines/mm) obtained at an energy of 8.93 μJ/cm^2^. (c) Surface profile of the planar grating.

**Table 1:** Comparison of Different SC-DFB Devices for Organic Lasing^a^

material	period (nm)	λ_em_ (nm)	*J*_th_^op^ (μJ/cm^2^)	fwhm (nm)	reference
BPFTT	140	612	2.41	0.07	^b^
BDPV2T	351	533	122	0.08	^35^
BP3T	140	615	2	0.22	^6^
BP3T	350	570	100	0.30	^36^
BSB-Me	330	475	10	0.90	^37^
BP2T	320	566	19	1.10	^38^
BP2T	800	565	25	1.50	^39^
BP3T	270	560	407	2.00	^34^

a λ_em_ is the emission wavelength and *J*_op_^th^ is the optical pumping threshold.

b Present work.

Finally, BPFTT is highly stable under atmospheric conditions. Over the course of ten months, only minimal variation in optical properties was observed in different measurements. However, we have observed a significant reduction in grating depth of approximately 14 nm in eight months following the fabrication of the planar DFB pattern (SI Section 15). This indicates the necessity of further encapsulation. Future work is planned to address this and to investigate the system’s electrically driven lasing properties.

## 3 Conclusions

In conclusion, a furan-substituted thiophene/phenylene co-oligomer was synthesized using a coupling reaction. The material was grown as a single crystal using PVT and analyzed for its suitability toward organic lasing. Structural analysis indicates the role of molecular planarity in the optoelectronic properties of this material. Lower MPP and SDP values, along with excited-state analysis, indicate the presence of strong vibrational coupling and nonradiative decay paths in the system. The fabricated devices exhibit moderate ambipolar mobility of 0.54 (hole) and 0.03 (electron) cm^2^ V^–1^ s^–1^, respectively, and optically pumped lasing thresholds of 15 and 17 μJ/cm^2^ for the 0–1 and 0–2 bands, respectively. The system characteristics are improved by using a distributed feedback resonator, and it has been shown that a nearly 7-fold reduction in optical pumping threshold and a 4-fold reduction in desired current density were obtained. Introducing contact engineering approaches can further improve the system to an electrically driven laser.

## 4 Methods

SCs are grown by using the PVT method in a high-purity Ar stream. A sublimation temperature of 340 °C with a 2.5 °C cm^–1^ variation in temperature profile is used for the crystal growth. Single-crystal X-ray diffraction analysis was carried out using Rigaku Oxford diffraction. ORCA 5.0.3 was used for density functional theory calculations.^40,41^ Geometry optimization and excited-state calculations were performed at the B3LYP/def2-SVP level of theory.^42^ Optimized geometries were used for the MPP and SDP calculations using the implementation available in the Multiwfn module.^43^ A detailed account is available elsewhere.^44^ Reorganization energy and rate constants were calculated using the excited-state dynamics (ESD) module available in ORCA.^45^ The FC and HT contributions were obtained directly from the ESD output file. A 405 nm diode laser was used for collecting PL and polarization-dependent PL. For polarization-dependent emission, a half-wave plate was introduced in front of the laser, and the polarization angle varied gradually. The corresponding variation in the output was monitored and measured by using a spectrometer (Ocean Optics HR2000+) aligned perpendicular to the emitting edge of the crystal. The absorption spectrum was collected using a BPFTT thin film with a thickness of 150 nm deposited by thermal evaporation at a pressure of ∼10^–5^ Pa. A Riken photoelectron yield spectrometer (AC-2) was used for the collection of PYS data. PLQY values are estimated using the integrating sphere method (Hamamatsu Photonics C9920-02). Mass spectra are measured by using the APCI ionization method in a Bruker Solarix XR.

For the fabrication of the OLETs, a p^++^-Si wafer with 300 nm SiO_2_ was cut into 1 cm^2^ pieces and used as the substrates. A polystyrene (PS) layer with a thickness of 25 nm was spin-coated on top of the substrates and annealed at 70 °C overnight. The SC with a thickness of ∼500 nm is electrostatically laminated on top of the PS layer. Heterostructure Ca and Au contacts are deposited by thermal evaporation with a pressure of ∼10^–7^ Torr. A Keysight B1500A parametric analyzer was used for the analysis of transistor characteristics. All of the transistor measurements were carried out inside a glovebox.

A nanosecond pulsed UV laser (Stanford Research System NL100) emitting 337 nm (13.8 Hz) was used as the excitation source for optical pumping studies (SI. Section 8). A stripe-shaped beam was focused on top of the crystal, and the emission was obtained through the crystal edges and collected by using a spectrometer (Andor SR-500i-A). The net gain coefficient for the samples is calculated using the variable stripe length method.^46^ The gain coefficient calculated for different pump energies is extrapolated to obtain the loss coefficient.^47^ A commercial nanopattern stamp on polyethylene terephthalate with a 140 nm period and a depth of 100 nm was used for thermal nanoimprinting of the DFB pattern. A 500 nm thick PMMA was spin-coated on top of a p^++^-Si wafer with 300 nm SiO_2_ and used for imprinting. The SC with a thickness of ∼700 nm was then laminated on top of the pattern.

## Supplementary Material



## Notes

Mass spectrum, note on MPP and SDP, degree of linear polarization calculation, Tauc plot and energy gap calculation, note on photophysical process, fluorescence lifetime calculation, hole–electron analysis for S_2_, S_3_, S_5_, and S_9_ states, optical pumping and VSL setups, gain and loss coefficient calculation, crystal dimensions, thickness-dependent gain narrowing profiles, stimulated emission cross-section calculation, coherence analysis, DFB lasing characteristics, DFB degradation, current density calculation, and OLET threshold calculation (PDF)
