# Supplementary material for: Structural, Photophysical, and Distributed Feedback Lasing Characteristics of Furan-Substituted Thiophene/Phenylene Co-Oligomer Single Crystals
Source: ACS Appl Mater Interfaces. 2025 Jun 16;17(26):38297–304. doi: 10.1021/acsami.5c05119 (PMC12232263; doi:10.1021/acsami.5c05119)
Supplement: Supplementary file 1 [file am5c05119_si_001.pdf]

Supporting Information

Structural, Photophysical and Distributed Feedback Lasing Characteristics of Furan Substituted Thiophene/Phenylene Co-Oligomer Single Crystals

Periyasamy Angamuthu Praveen,[*,†] Thangavel Kanagasekaran,[*,‡] Chaoyan Ma,[¶] Masahiro Terada,[¶] Tienan Jin,[§] Yusuke Wakabayashi,[†] and Hidekazu Shimotani[*,†]

†Department of Physics, Graduate School of Science, Tohoku University, Sendai - 980-8578, Japan

‡Organic Optoelectronics Laboratory, Department of Physics, Indian Institute of Science Education and Research - Tirupati, Tirupati - 517619, India

¶Department of Chemistry, Graduate School of Science, Tohoku University, Sendai - 980-8578, Japan

§Research and Analytical Center for Giant Molecules, Graduate School of Science, Tohoku University, Sendai - 980-8578, Japan

E-mail: praveen@tohoku.ac.jp; kanagasekaran@iisertirupati.ac.in; shimotani@tohoku.ac.jp

Contents

1. Mass spectrum **2**

2. MPP and SDP **2**

3. Degree of linear polarization (DOLP) **2**

4. Tauc Plot and Energy gap calculation **3**

5. Photophysical process **3**

6. Fluorescence lifetime **4**

7. Hole-electron Analysis **4**

8. Optical pumping and VSL setups **4**

9. Gain and loss coefficient calculation **5**

10. Crystal dimensions **6**

11. Thickness dependent gain narrowing profiles **6**

12. Stimulated emission cross-section **7**

13. Coherence **7**

14. DFB Lasing Characteristics **7**

15. DFB degradation **8**

16. Current density calculation **8**

17. OLET threshold calculation **9**

1. Mass spectrum

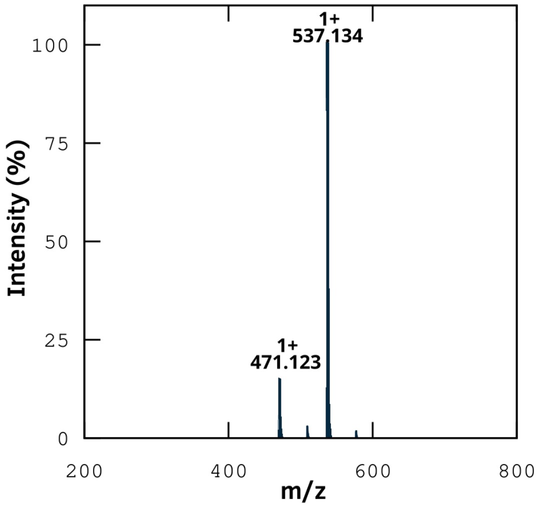

Figure S1: Atmospheric pressure chemical ionisation (APCI) based mass spectra of BPFTT system. The chemical formula for BPFTT is $C_{36}H_{24}S_2O$. Considering the mass of each atom as C: 12.01 Da, H: 1.01 Da, S: 32.07 Da, O: 16.00 Da, the observed peak at 537.13 Da confirms the BPFTT's atomic composition. The minor peak at 471.12 Da suggests the presence of fragment ion of BPFTT due to loss of furan or aryl group. However, the data is recorded for the as-synthesised compound, and the role of these impurities in optoelectronics characteristics is minimal since the crystals are grown using a three-zone furnace with a two-time sublimed material.

2. MPP and SDP

Using the least squares fitting method, the idea is to fit all the atoms in a molecule to a fitting plane. Once the atoms are fitted to a plane, MPP can be measured as the root mean square deviation of atoms to the fitting plane. SDP can be calculated as the difference in atomic distance to a fitting plane. So, MPP can measure an overall deviation of atoms, and SDP indicates the molecular span relative to the fitting plane. The molecular planarity indices are calculated for the gas phase optimized geometry without any symmetry constraints.

3. Degree of linear polarization (DOLP)

The DOLP measurements were carried out by exciting a BPFTT single crystal with a linearly polarized continuous-wave laser (405 nm, 5 mW) incident normal to the substrate surface, i.e., along the crystallographic c-axis. The polarization angle of the excitation light within the ab-plane (parallel to the substrate) was controlled using a half-wave plate. The 0° polarization angle was defined as the condition where the electric field of the excitation light is parallel to the crystal's b-axis (long axis). Photoluminescence emitted from the bc-face (side edge of the crystal) was collected into an optical fiber for intensity measurements. The results showed a strong dependence of the PL intensity on the excitation light's polarization angle. This angular dependence clearly indicates a slight tilt of the transition dipole moment from the c-axis, resulting in an in-plane component that efficiently interacts with the polarized excitation light. The maximum emission

intensity was observed at an excitation polarization angle of -20°, implying that the in-plane component of the transition dipole moment is oriented approximately 20° from the b-axis toward the a-axis.

DOLP values are calculated using the relation,

$$DOLP = \frac{I_{max} - I_{min}}{I_{max} + I_{min}} \tag{S.1}$$

Here I_{max} and I_{min} are maximum and minimum intensities obtained from the polarization dependent PL spectrum.

4. Tauc Plot and Energy gap calculation

To calculate the frontier orbital gap (E_g), Tauc plots ($(\alpha h\nu)^2$ as a function of photon energy) are used. Here α is the absorption coefficient, obtained by dividing the absorbance with sample thickness, h is Planck's constant and ν is the photon frequency. Extrapolating the linear slope of $(\alpha h\nu)^2$ to zero absorption coefficient yield the E_{opt} value. E_{HOMO} values are obatined from the PYS spectra. Using the relation, $\Delta_{ex} = E_{opt}/3$ [1], exciton binding energy value is obtained. E_{LUMO} values are obtained by $E_{LUMO} = E_{HOMO} + E_{opt} + \Delta_{ex}$.

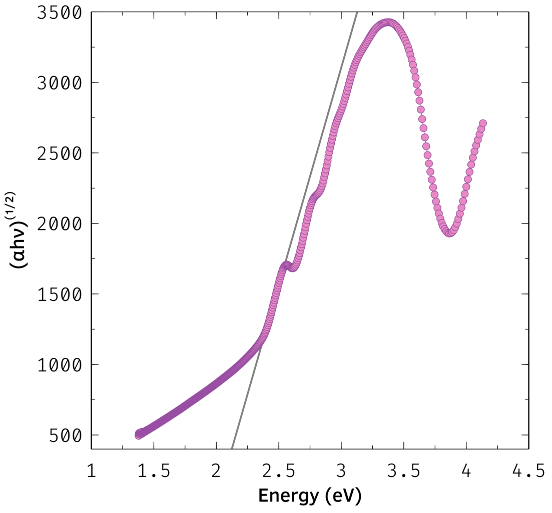

Figure S2: Tauc plot to calculate E_{opt} in BPFTT thin films.

5. Photophysical process

In a four-level system, the fluorescence quantum yield can be given as

$$\Phi_f = \frac{k_f}{k_f + k_{nr}} \tag{S.2}$$

where k_f is the fluorescence rate constant and k_{nr} is the non-radiative process. These two parameters can be further related by the fluorescence lifetime τ_f as $\tau_f = (k_f + k_{nr})^{-1}$. By combining the two relations, the k_f and k_{nr} can be described as $k_f = \Phi_f/\tau_f$ and $k_{nr} = (1 - \Phi_f)/\tau_f$.

6. Fluorescence lifetime

Strickler - Berg relation is one of the widely used empirical relations to estimate the fluorescence rate constant [2,3]. It relates the absorption coefficient and fluorescence of an emissive system as,

$$k_f = 1.511 \times 10^3 \frac{\int I(E)dE}{\int E^{-3} I(E)dE} n^2 \frac{g_1}{g_u} \int \epsilon_m(E) dlnE \tag{S.3}$$

Here, I(E) denotes the fluorescence spectrum, n is the refractive index, ϵ_m is the molar extinction coefficient, $g_{1,u}$ are degeneracies of the lower and upper states, and for fluorescence $g_1/g_u = 1$. The equation can be further simplified by introducing the oscillator strength f and by removing the spectral shapes and solvent effects as [3],

$$k_f = 4.34 \times 10^7 \frac{E_f^3}{E_A} n^2 f \tag{S.4}$$

Where E_f and E_A are the vertical energies from PL and absorption spectrum, and the oscillator strength f can be obtained from the relation $f = 3.843 \times 10^{-5} \int \epsilon_m(E)dE$. The molar extinction coefficient ϵ_m can be calculated from the absorption spectrum as $\epsilon_m = \frac{\alpha\lambda}{4\pi}$.

It is worth noting that the accuracy of the relation is not limited to the simplifications; rather, it arises due to the inconsistencies in experimental measurements. In particular, errors related to fluorescence and PLQY measurements significantly affect the accuracy [4,5].

7. Hole-electron Analysis

The hole and electron analysis was also performed for the S_2, S_3, S_5 and S_9 states. However, their overall contribution is very less compared to the S_1 state. For example the oscillator strength f_{osc} of S_1 is about 2.61 (arb) and the corresponding values for the states S_2, S_3, S_5 and S_9 are 0.08, 0.19, 0.26 and 0.16, respectively.

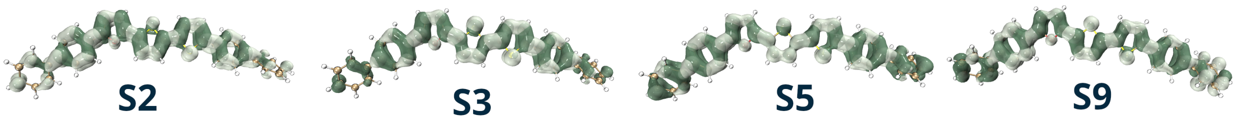

Figure S3: Hole-electron distribution in S_2, S_3, S_5 and S_9 states of BPFTT.

8. Optical pumping and VSL setups

A stripe shaped beam is used for the optical pumping measurements. The width of the stripe is 60 μm and the length is 4 mm. Wavelength of the laser is 337 nm with a pulse duration of 3.5 ns and the peak intensity within the stripe short axis profile (without any ND filter) is 35.29 mJ/cm^2.

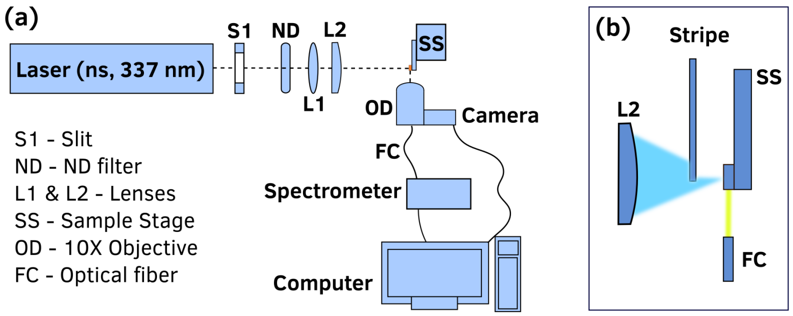

Figure S4: (a) Optical pumping and (b) VSL setups

9. Gain and loss coefficient calculation

The net gain coefficient (g) was calculated using variable stripe method (VSL). A thin copper plate was introduced between the sample and L2. The length of the beam is adjusted gradually by moving the copper plate with constant beam width. The variation in output intensity (I) with respect to the beam length (L) was recorded using a spectrometer. The g was calculated by fitting the data to the relation [6,7]

$$I = I_s(e^{gL} - 1)/g \tag{S.5}$$

Here, I_s is the intensity of spontaneous emission. The loss coefficient (k) does not depends on pump intensity. So, it can be calculated by plotting g for different pump intensities, like below the threshold, at threshold and above threshold and extrapolating it to $I_s = 0$, where $g = -k$.

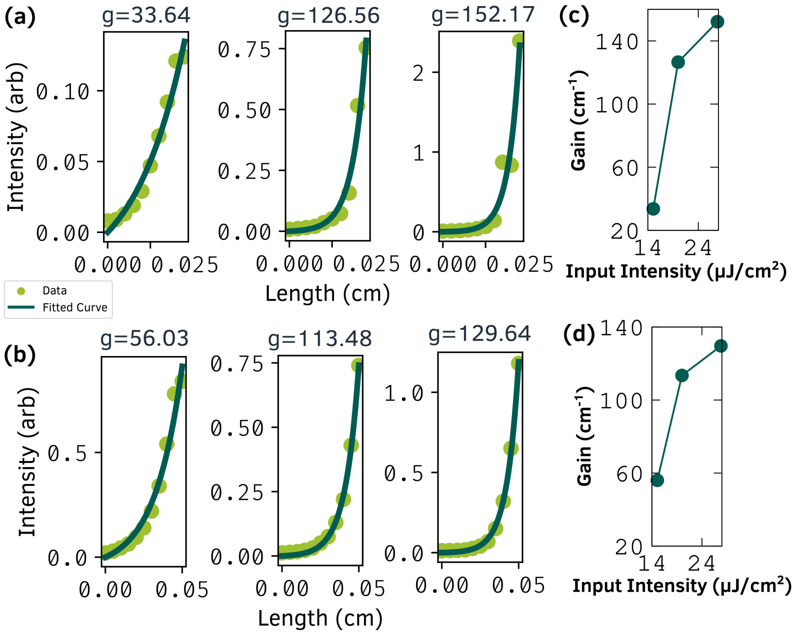

Figure S5: The g values calculated for (a) 0-1 band (b) 0-2 band with the pump intensities 15, 20 and 27 $\mu J/cm^2$, respectively. (c) Corresponding loss coefficients for (c) 0-1 and (0-2) bands.

10. Crystal dimensions

By tweaking the crystal growth process, the dimensions of the crystals can be varied. Even a single growth would contain crystals of different sizes. The crystal widths are measured using an inbuilt camera and the program in the Lecia microscope system. The thickness of the crystals is measured using an AFM profiling through the substrate to the edge of the crystal.

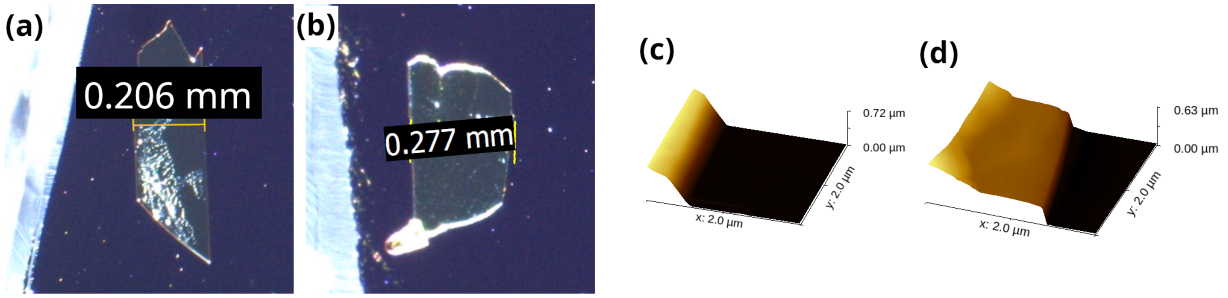

Figure S6: (a-b) Crystal widths measured using microscope (c-d) Thickness profiles of some crystals using AFM.

11. Thickness dependent gain narrowing profiles

The optically pumped lasing behaviour of BPFTT SCs can be broadly classified into three categories depending on their thickness. Often, crystals with less than 600 nm thickness exhibit 0-1 band gain narrowing profiles. On the other hand, crystals with a thickness of more than 1000 nm favour the 0-2 band. Crystals with thicknesses between 600 and 1000 nm often exhibit dual gain narrowing phenomena, i.e., both 0-1 and 0-2 bands amplify with respect to the input intensity.

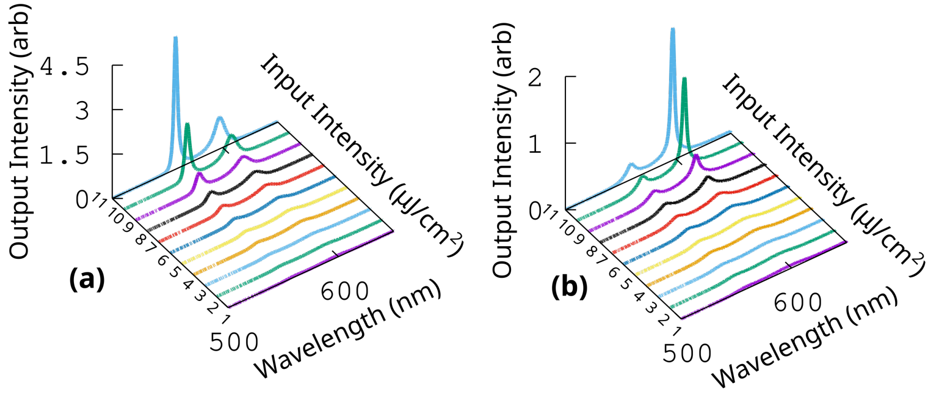

Figure S7: Emission profile in BPFTT SCs with thickness (a) 600 nm and (b) 800 nm. The y-axis corresponds to pump intensity as (in the order of 1 to 11) 4.78, 6.38, 7.12, 8.93, 10.45, 15.08, 16.10, 20.01, 23.40, 27.8, 32.6 $\mu J/cm^2$, respectively.

12. Stimulated emission cross-section

By using the fluorescence spectrum and the lifetime values, the stimulated emission cross-section can be calculated as [8],

$$\sigma(\lambda) = \frac{\lambda^4 \Phi_f}{8\pi n^2(\lambda) c \tau_f} \tag{S.6}$$

Where Φ_f is the PLQY, c is the velocity of light, n is the effective refractive index calculated using [9],

$$n = \frac{\lambda^2}{2\Delta\lambda L} \tag{S.7}$$

13. Coherence

The longitudinal spatial coherence (L_c) length is calculated using $L_c = \lambda^2/2\pi\Delta\lambda$. From this, the temporal coherence value is calculated using $\tau_c = L_c/c$ [10,11]. Here λ is the emission wavelength, $\Delta\lambda$ is the FWHM and c is the speed of light in vacuum.

14. DFB Lasing Characteristics

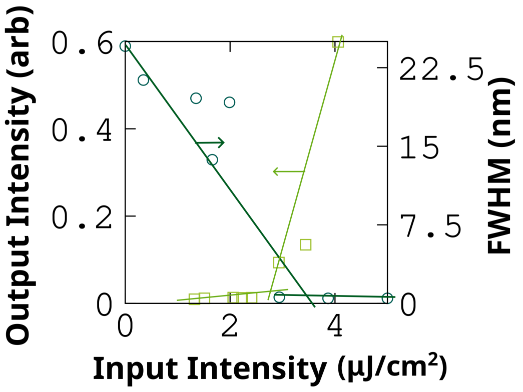

Figure S8: Gain narrowing curve for DFB device

15. DFB degradation

After eight months, significant decrease in grating depth of approximately 14 nm has been observed. Since the samples were kept under atmospheric conditions, moisture absorption and viscoelastic relaxation may have played a role in reducing surface energy and smoothing the surface. [12,13]

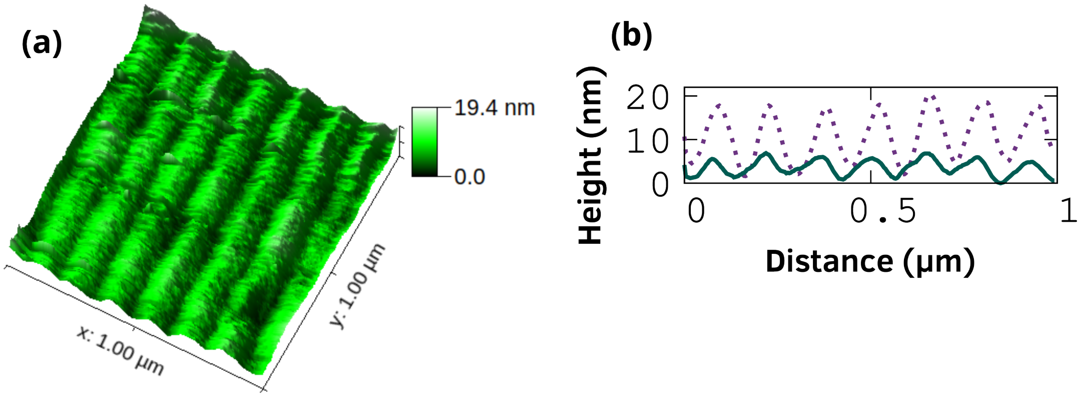

Figure S9: (a) AFM image of the DFB grating after eight months. (b) Line profiles extracted from the AFM image for the fresh sample (dashed purple line) and the sample after eight months (solid green line).

16. Current density calculation

Threshold current density are calculated using the realtion [14],

$$J_{th} = \frac{N_{th} \times Q \times W_{rz}}{\chi \times \tau} \tag{S.8}$$

where, J_{th} is the threshold current density, q is the elementary charge, W_{rz} is the width of the recombination zone, typically 1 μm for BPFTT OLETs, χ is the singlet generation ration (0.25), N_{th} is population of excitons at stimulated emission and τ is the radiation lifetime.

The N_{th} is calculated using,

$$N_{th} = \frac{\eta \times P_{th}}{z \times (\frac{h \times c}{\lambda_e})} \tag{S.9}$$

where $\eta = 1 - exp(-\alpha)$ is the absorption efficiency (0.89 for BPFTT) and α is the absorption coefficient, P_{th} is the optical pumping threshold, z is the cavity thickness, h is Planck's constant, c is speed of light in vacuum and λ_e is the excitation wavelength.

The radiation lifetime is calculated using the relation [15], $\tau = 1.499/f_{osc}E^2$, where f_{osc} is the oscillator strength and E is the excitation energy.

17. OLET threshold calculation

The device thresholds are calculated by plotting $\sqrt{I}_d$ vs V_g and by extrapolating the linear fit to $\sqrt{I}_d = 0$.

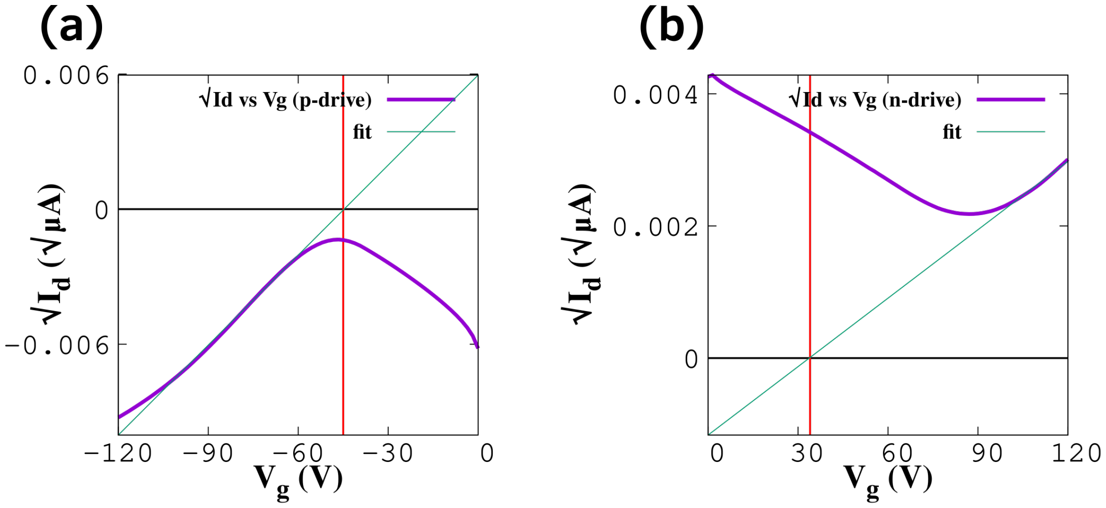

Figure S10: $\sqrt{I}_d$ vs V_g curves of (a) p and (b) n-drive. The red line indicates the threshold and black line is $\sqrt{I}_d = 0$.

References

1. Bredas, J.L. “Mind the gap!” Materials Horizons 1 (2014): 17–19.
2. Strickler, S. J., and Robert A. Berg. “Relationship between absorption intensity and fluorescence lifetime of molecules.” The Journal of chemical physics 37.4 (1962): 814-822.
3. Gierschner, Johannes, et al. “Luminescence in crystalline organic materials: from molecules to molecular solids.” Advanced Optical Materials 9.13 (2021): 2002251.
4. Hirata, Shuzo, et al. “Highly efficient blue electroluminescence based on thermally activated delayed fluorescence.” Nature materials 14.3 (2015): 330-336.
5. Shi, Junqing, et al. “Solid state luminescence enhancement in π-conjugated materials: unraveling the mechanism beyond the framework of AIE/AIEE.” The Journal of Physical Chemistry C 121.41 (2017): 23166-23183.
6. Mizuno, H., et al. “Optically pumped lasing from single crystals of a cyano-substituted thiophene/phenylene co-oligomer.” Advanced Optical Materials 2 (2014) 529–534.
7. Munoz-Marmol, R., et al. “Influence of blending ratio and polymer matrix on the lasing properties of perylenediimide dyes.” The Journal of Physical Chemistry C 122 (2018) 24896–24906.
8. Dong, Haiyun, et al. “Dual-wavelength switchable vibronic lasing in single-crystal organic microdisks.” Nano letters 17.1 (2017): 91-96.
9. Miura, Taiki, et al. “Laser oscillation of an organic distributed-feedback laser at the edge of a mini stopband.” Applied Physics Express 14.5 (2021): 052007.
10. Oliveira, Vitor, et al. “Laser surface patterning using a Michelson interferometer and femtosecond laser radiation.” Optics & Laser Technology 44.7 (2012): 2072-2075.
11. Marshall, Graham D., et al. “Coherence properties of a single dipole emitter in diamond.” New Journal of Physics 13.5 (2011): 055016.
12. Ito, Asae, Arisa Shin, and Koh-hei Nitta. “Viscoelastic Properties of Water-Absorbed Poly (methyl methacrylate) Doped with Lithium Salts with Various Anions.” Molecules 27.20 (2022): 7114.
13. Vogtmann, Dana E. Stress relaxation in poly (methyl methacrylate)(PMMA) during large-strain compression testing near the glass transition temperature. Diss. The Ohio State University, 2009.
14. Maruyama, Kenichi, et al. “Ambipolar light-emitting organic single-crystal transistors with a grating resonator.” Scientific Reports 5.1 (2015): 10221.
15. Bhattacharya, Arka, et al. “Theoretical insights on pyrene end-capped thiophenes/furans and their suitability towards optoelectronic applications.” Computational and Theoretical Chemistry 1225 (2023): 114135.
